# Verapamil Targets Membrane Energetics in Mycobacterium tuberculosis

**DOI:** 10.1128/AAC.02107-17

**Published:** 2018-04-26

**Authors:** Chao Chen, Susana Gardete, Robert Sander Jansen, Annanya Shetty, Thomas Dick, Kyu Y. Rhee, Véronique Dartois

**Affiliations:** a Public Health Research Institute, New Jersey Medical School, Rutgers, The State University of New Jersey, Newark, New Jersey, USA; b Weill Cornell Medical College, Weill Department of Medicine, New York, New York, USA; c Department of Medicine, Yong Loo Lin School of Medicine, National University of Singapore, Singapore; d Department of Medicine, New Jersey Medical School, Rutgers, The State University of New Jersey, Newark, NJ, USA

**Keywords:** Mycobacterium tuberculosis, efflux pump, bioenergetics, verapamil

## Abstract

Mycobacterium tuberculosis kills more people than any other bacterial pathogen and is becoming increasingly untreatable due to the emergence of resistance. Verapamil, an FDA-approved calcium channel blocker, potentiates the effect of several antituberculosis (anti-TB) drugs *in vitro* and *in vivo*. This potentiation is widely attributed to inhibition of the efflux pumps of M. tuberculosis, resulting in intrabacterial drug accumulation. Here, we confirmed and quantified verapamil's synergy with several anti-TB drugs, including bedaquiline (BDQ) and clofazimine (CFZ), but found that the effect is not due to increased intrabacterial drug accumulation. We show that, consistent with its *in vitro* potentiating effects on anti-TB drugs that target or require oxidative phosphorylation, the cationic amphiphile verapamil disrupts membrane function and induces a membrane stress response similar to those seen with other membrane-active agents. We recapitulated these activities *in vitro* using inverted mycobacterial membrane vesicles, indicating a direct effect of verapamil on membrane energetics. We observed bactericidal activity against nonreplicating “persister” M. tuberculosis that was consistent with such a mechanism of action. In addition, we demonstrated a pharmacokinetic interaction whereby human-equivalent doses of verapamil caused a boost of rifampin exposure in mice, providing a potential explanation for the observed treatment-shortening effect of verapamil in mice receiving first-line drugs. Our findings thus elucidate the mechanistic basis for verapamil's potentiation of anti-TB drugs *in vitro* and *in vivo* and highlight a previously unrecognized role for the membrane of M. tuberculosis as a pharmacologic target.

## INTRODUCTION

Tuberculosis (TB) now surpasses AIDS as the leading cause of death from a single infectious agent (1), and clinical resistance has emerged to every anti-TB drug in use today, including bedaquiline, which was launched against multidrug-resistant (MDR) TB only 4 years ago. New or repurposed drugs with novel mechanisms of action, including agents that potentiate existing drug regimens, are needed.

Verapamil (VER) was developed as a calcium channel blocker to treat hypertension (2) but is also a substrate and inhibitor of P-glycoprotein (P-gp), a mammalian drug efflux protein (3, 4), and of uptake transporters such as the organic cation transporter (OCT) protein (5). When used against Mycobacterium tuberculosis, verapamil was found to potentiate the activity of bedaquiline and clofazimine (6). Against drug-resistant M. tuberculosis isolates, verapamil was shown to partially restore the potency of rifampin (RIF) (7, 8), isoniazid (INH) (9), ethambutol (10), and the fluoroquinolones (7, 11–13). Because verapamil is an inhibitor of P-glycoprotein, these anti-TB activities are generally assumed to be due to the inhibition of efflux pumps that are either constitutive or induced upon acquisition of drug resistance (14, 15). Verapamil was also shown to restore the acquisition of drug tolerance inside macrophages, a phenomenon that was again attributed to bacterial efflux pumps (16, 17) whose expression is induced upon macrophage infection (18).

In mice, addition of verapamil to the first-line drug regimen accelerated the sterilization of infected lungs (19). These observations suggested that verapamil could potentiate anti-TB drugs in the clinic (15), leading to the initiation of at least one clinical trial (Annual Report of the National Institute for Research in Tuberculosis; http://www.nirt.res.in).

Despite the widespread belief that the effect of verapamil on M. tuberculosis is due to the drug's interference with bacterial efflux pumps, formal demonstration of increased intracellular drug concentrations in the presence of verapamil has been lacking, and the direct contribution of specific efflux pumps to the extrusion of anti-TB drugs has not been demonstrated.

Here we characterize the mechanisms underlying verapamil-mediated potentiation of anti-TB drugs. We first test the hypothesis that verapamil modulates the intracellular concentrations of anti-TB drugs in drug-sensitive as well as drug-resistant M. tuberculosis bacilli and in macrophages. We next show that the drug causes a collapse of the membrane potential of M. tuberculosis, providing a possible explanation for the reported synergy of verapamil with anti-TB drugs that target or required oxidative phosphorylation and validating the identification of the mycobacterial membrane as a therapeutic target for anti-TB drug discovery.

## RESULTS

**FIG 1:**
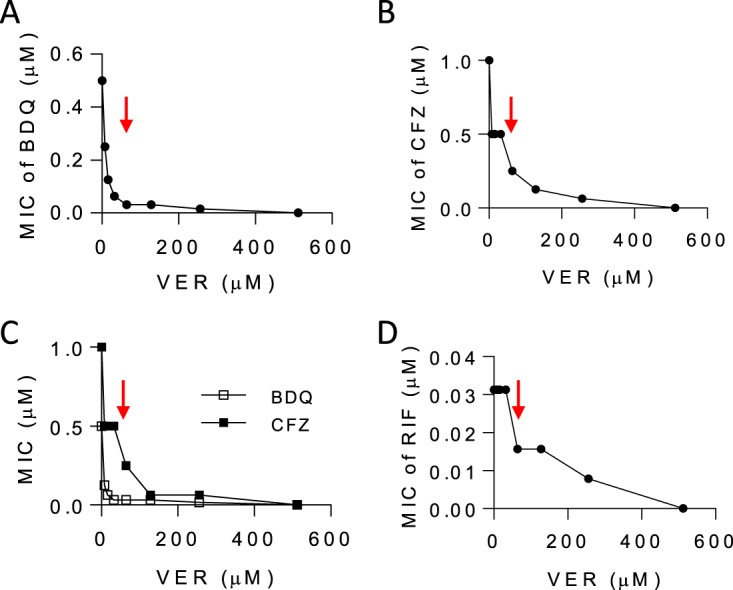
Pharmacodynamic interactions between verapamil and anti-TB drugs. (A to C) Verapamil (VER) was tested in combination with bedaquiline (BDQ) and clofazimine (CFZ) in checkerboard assays against wild-type M. tuberculosis H37Rv (A and B) as well as against multidrug-resistant M. tuberculosis strain R543 (resistant to rifampin, isoniazid, ethambutol, and pyrazinamide) (7) (C). The MIC of bedaquiline alone and the MIC of clofazimine alone against H37Rv and R543 were identical at 0.5 and 1.0 μM (0.28 and 0.47 mg/liter), respectively. (D) Efficacy of the verapamil-rifampin (RIF) combination. The MIC of RIF alone was 0.032 μM (0.025 mg/liter). The FICI values (MIC_drugAB_/MIC_drugA_ + MIC_drugBA_/MIC_drugB_) were 0.06 and 0.19 (synergistic) for bedaquiline and clofazimine, respectively, and 0.75 (additive) for rifampin. The red arrows point to the verapamil concentration corresponding to one-fourth its MIC (128 μM [58 mg/liter]). Each experiment included technical duplicates and was performed three times independently. Data from one representative experiment are shown in each panel.

To confirm and quantify the *in vitro* potentiation and growth-inhibitory effects of verapamil, we performed dose-response drug combination experiments, using drug-susceptible and MDR TB strains. The MIC of verapamil against wild-type M. tuberculosis H37Rv and MDR strain R543 was 512 μM. We observed that verapamil synergized with the oxidative-phosphorylation-targeting drugs bedaquiline (F_1_F_o_ ATP-synthase [20]) and clofazimine (NADH dehydrogenase II [21]). At one/fourth its MIC (128 μM), verapamil caused 20-fold and 4-fold decreases in the bedaquiline and clofazimine MICs, respectively (Fig. 1A and B), similarly to the previously reported 8-fold potentiation observed at comparable verapamil concentrations (6). Potentiation by verapamil was observed at concentrations as low as 8 μM (1/64 of its MIC) in both drug-susceptible and multidrug-resistant M. tuberculosis strains (Fig. 1A to C). In addition, verapamil potentiated rifampin in an additive manner (Fig. 1D).

**FIG 2:**
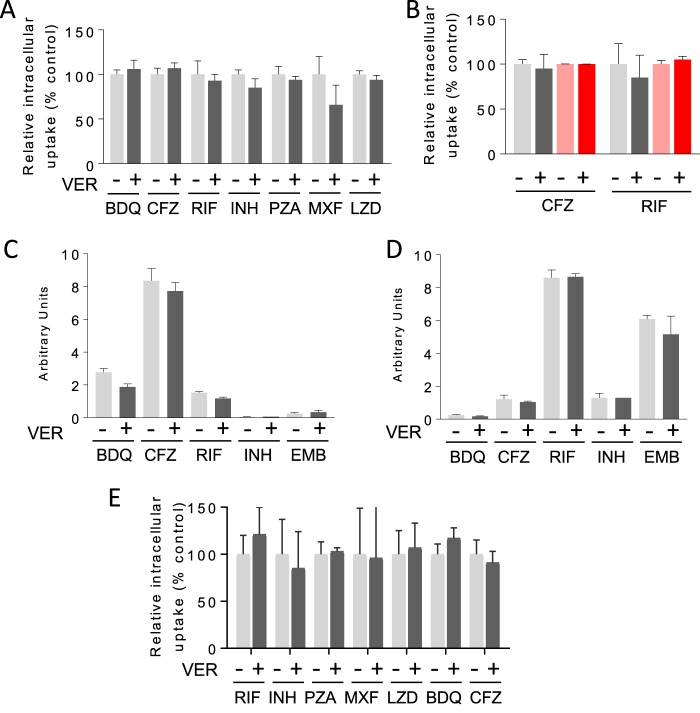
Effect of verapamil on the intracellular concentration of anti-TB drugs in M. tuberculosis bacilli and in THP-1 macrophages. (A) Intracellular accumulation of anti-TB agents in exponential-phase M. tuberculosis Δ*panCD ΔleuD* (an attenuated strain that is noninfectious to mammals for use in biosafety level 2 [BSL2] settings). All drugs were supplemented at 4-fold their respective MIC values with or without verapamil at one/fourth its MIC (128 μM). (B) Intracellular accumulation of rifampin and clofazimine in wild-type (gray shades) and multidrug-resistant (red shades) R543 M. tuberculosis. (C and D) Effect of verapamil on the uptake of drugs by M. tuberculosis in solid-phase culture after 24 h of incubation at one/fourth its MIC: data corresponding to the recovery of bacterially associated drug (C) and drug remaining in media (D) with and without coincubation with verapamil are shown. (E) Intracellular accumulation of anti-TB drugs in uninfected human THP-1 macrophages. RIF, rifampin; INH, isoniazid; EMB, ethambutol; PZA, pyrazinamide; MXF, moxifloxacin, LZD, linezolid; BDQ, bedaquiline; CFZ, clofazimine. Values are means (*n* = 3) ± standard errors. Each experiment included technical duplicates and was performed three times independently. Data from one representative experiment are shown in each panel. Two-tailed unpaired *t* tests were performed to compare uptake data determined in the presence and absence of verapamil. All *P* values are >0.05 except for those corresponding to RIF and BDQ in panel C (*P* = 0.0054 and 0.0078, respectively, with lower intrabacterial uptake in the presence of verapamil).

As it is generally believed that verapamil acts as an inhibitor of mycobacterial efflux pumps (14, 15), we directly measured intrabacterial drug concentrations in M. tuberculosis by high-performance liquid chromatography (HPLC)-coupled tandem mass spectrometry (LC/MS-MS). However, pretreatment with verapamil failed to yield increased intrabacterial concentrations of any of the anti-TB drugs tested (Fig. 2A; see also Fig. S1 in the supplemental material). We similarly failed to observe increased drug concentrations in an MDR M. tuberculosis strain (R543) previously reported to exhibit increased levels of efflux pump expression and drug synergies with verapamil (7) (Fig. 2B). Given the lack of a suitable positive control, i.e., a compound experimentally demonstrated to increase the intramycobacterial concentration of any anti-TB drug, we sought to validate our findings using an orthogonal culture system and an LC/MS-based assay (22). In this system, M. tuberculosis bacteria grow as a confluent colony atop a filter support and retain the native architecture of their outer envelope, normally removed by the detergents used to support planktonic growth in broth. This culture system enables rapid and efficient recovery of both intra- and extracellular drug molecules owing to the physically discrete separation of bacterial cells from their extracellular environment. This approach also excludes the possibility of sample handling-associated drug leakage from M. tuberculosis cells. This system similarly failed to demonstrate increased intrabacterial levels of a range of drugs following coincubation with verapamil (Fig. 2C and D). These data confirm the negative results obtained in liquid cultures and indicate that the potentiating effect of verapamil with anti-TB drugs is not attributable to their accumulation within M. tuberculosis.

Verapamil has also been shown to accelerate the clearance of M. tuberculosis bacilli in mouse lungs by standard anti-TB therapy (19). To determine whether antitubercular drugs accumulate in phagocytic cells to a greater extent in the presence of verapamil, drug uptake assays were performed in human THP-1 macrophages. Preincubation with verapamil failed to increase the intramacrophage concentration of a range of first- and second-line antituberculosis drugs (Fig. 2E). Similar results were obtained in activated murine bone marrow-derived macrophages (data not shown).

**FIG 3:**
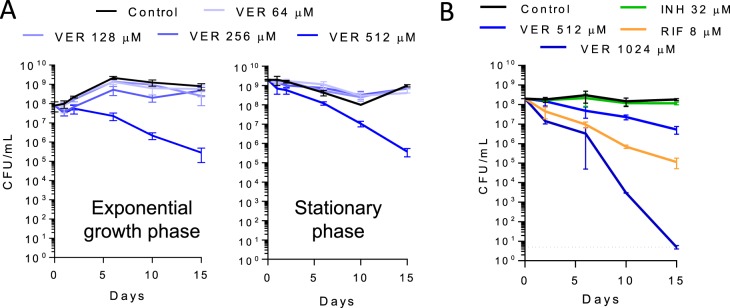
Time- and dose-dependent killing of (A) exponentially growing and stationary-phase and (B) nutrient-starved nonreplicating M. tuberculosis H37Rv by verapamil (MIC, 512 μM). The dotted line shows the detection limit (5 CFU/ml). In the nutrient starvation (Loebel) assay, rifampin (RIF) and isoniazid (INH) were used as positive and negative controls, at 8 and 32 μM or approximately 100-fold their respective 90% bactericidal concentrations (MBC_90_) against replicating M. tuberculosis (58). Each experiment included four technical replicates and was performed three times independently. Data from one representative experiment are shown in each panel.

**FIG 4:**
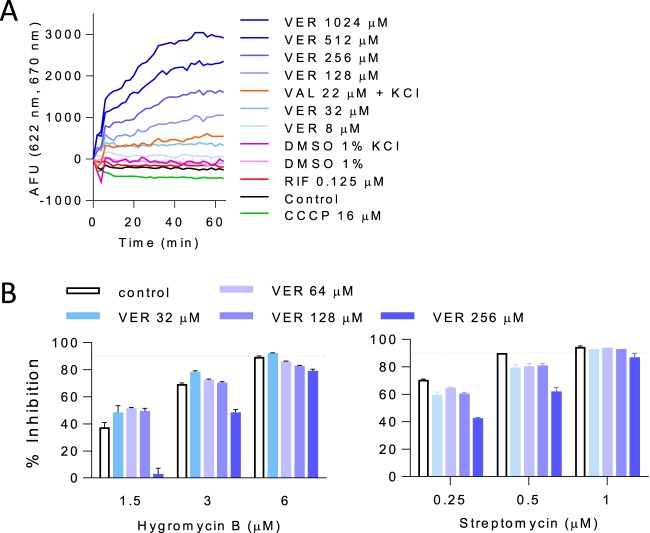
Effect of verapamil on membrane functions. (A) Dose-response effect of verapamil on M. tuberculosis membrane electric potential. Membrane depolarization was measured using the membrane potential-sensitive fluorescent DiSC_3_ dye (5), which concentrates in energized membranes such that high local concentrations lead to decreased fluorescence intensity due to quenching. Upon dissipation of the membrane potential, the dye is released in the extracellular space, resulting in increased fluorescence. VER, verapamil; VAL, valinomycin; RIF, rifampin; CCCP, carbonyl cyanide m-chlorophenyl hydrazine; AFU, arbitrary fluorescence units. (B) Effect of verapamil on the inhibitory activity of the aminoglycosides hygromycin B and streptomycin. The MIC of hygromycin B and that of streptomycin in the absence of verapamil were 6 and 0.5 μM, respectively.

To gain potential insight into verapamil's drug-potentiating activity, we next examined its intrinsic antimycobacterial activity. Interestingly, MIC and time-kill assays performed with exponentially growing and stationary-phase M. tuberculosis bacteria revealed a 3-log reduction of viable counts in exponentially growing and stationary-phase cultures after 15 days of verapamil exposure at its MIC. Moreover, this bactericidal activity was also observed with starved nonreplicating M. tuberculosis bacteria (Fig. 3), which exhibit a high level of tolerance of most drugs (23). Together, these findings suggested that verapamil's intrinsic antimycobacterial activity might be mediated by targeting a core process required for viability (rather than growth alone) whose partial inhibition might also mediate its drug-potentiating activity (24). Cationic amphiphiles, similarly to verapamil, have been reported to interact with the membrane and to cause structural and electrical perturbations that affect membrane potential (25, 26). Maintenance of an energized membrane is essential for viability of M. tuberculosis, independently of its physiological state (27). Previous studies have shown that disruption of membrane potential is lethal to both growing and nongrowing M. tuberculosis bacteria (23, 28, 29). We therefore hypothesized that verapamil may directly impact the membrane energetics of M. tuberculosis. Such a mechanism would be consistent with verapamil's ability to synergize with drugs affecting oxidative phosphorylation—a process depending on intact membrane function—whereas mostly additive effects were observed with other drugs (14, 17). To determine the effect of verapamil on mycobacterial membrane potential, we used the fluorescent potentiometric dye 3,3′-dipropylthiadicarbocyanine iodide [DiSC_3_(5)], which accumulates in polarized membranes and self-quenches, while depolarization results in dye release into the medium and dequenching of fluorescence. Treatment with verapamil caused a rapid and concentration-dependent collapse of the membrane electric potential in less than an hour (Fig. 4A). At concentrations of 32 μM and higher, this effect exceeded that induced by the valinomycin (VAL) control, a K^+^ ionophore which specifically dissipates the electric potential (ΔΨ) component of the proton motive force (PMF) in the presence of exogenous K^+^. Carbonyl cyanide m-chlorophenyl hydrazone (CCCP), a strong protonophore which specifically dissipates the transmembrane proton gradient (ΔpH) component of the PMF, caused a slight decrease in fluorescence. This might have been due to a compensatory increase in ΔΨ induced by the cells in order to maintain a constant PMF (30, 31). Such an increase in membrane potential further concentrates the DiSC_3_(5) dye in the membrane, such that high local concentrations lead to decreased fluorescence intensity due to further quenching (Fig. 4A). As the verapamil-induced membrane depolarization precedes loss of viability, which occurs within only days of exposure (Fig. 3), these results indicate that the apparent loss of membrane energetics induces, rather than accompanies, bacterial death.

To provide independent functional evidence of verapamil's ability to disrupt M. tuberculosis's membrane energetics, we exploited the fact that an energized membrane is required for the uptake of aminoglycosides in M. tuberculosis (32) and tested the effect of verapamil on aminoglycoside activity. As expected, the growth-inhibitory activity of the two aminoglycosides streptomycin and hygromycin (Hgr) B decreased in a verapamil-dependent manner (Fig. 4B). We then tested whether verapamil might act by affecting the physical integrity of M. tuberculosis's membrane, and showed that verapamil increased neither the release of a red fluorescent cytoplasmic protein nor the accumulation of Sytox green, a nonpermeable fluorescent dye (Fig. S2). These data thus collectively show that verapamil impacts membrane energetics without disrupting its physical integrity.

**FIG 5:**
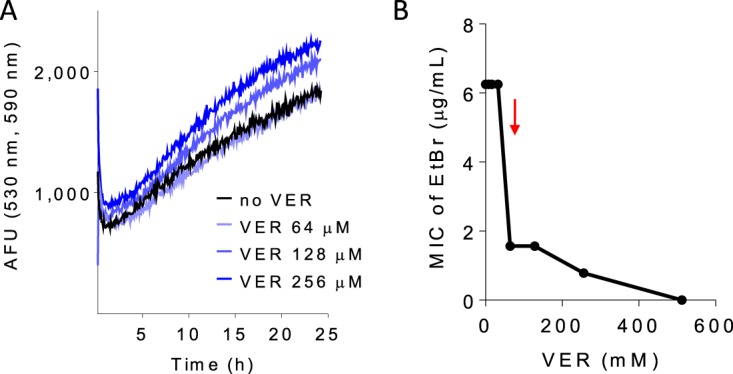
Effect of verapamil on ethidium bromide (EtBr) uptake and activity in M. tuberculosis H37Rv. (A) Dose response of verapamil on the uptake of EtBr measured by fluorescence at 590 nm (excitation wavelength,530 nm). (B) Pharmacodynamic interactions between verapamil and EtBr. The FIC index value (MIC_drugAB_/MIC_drugA_ + MIC_drugBA_/MIC_drugB_) was 0.25, indicating synergy. The red arrow points to the level corresponding to one/fourth the verapamil MIC (128 μM). Each experiment was performed three times independently. Data from one representative experiment are shown in each panel.

Ethidium bromide (EtBr) is considered a broad substrate of efflux pumps, most of which require the PMF (33, 34), and has been used as a probe to monitor the activity of efflux pumps in mycobacteria (35). Verapamil has been shown to affect the intracellular accumulation of EtBr in M. tuberculosis (36, 37), an effect generally attributed to direct inhibition of efflux pumps. However, dissipation of the PMF by verapamil could impair efflux, in turn leading to increased intracellular EtBr concentrations. Using assay conditions under which we found no effect on anti-TB drug uptake, we observed increased intrabacterial accumulation of EtBr in the presence of verapamil (Fig. 5A). Accordingly, verapamil and EtBr synergized against M. tuberculosis with a fractional inhibitory concentration index (FICI) value of 0.25 (Fig. 5B).

**FIG 6:**
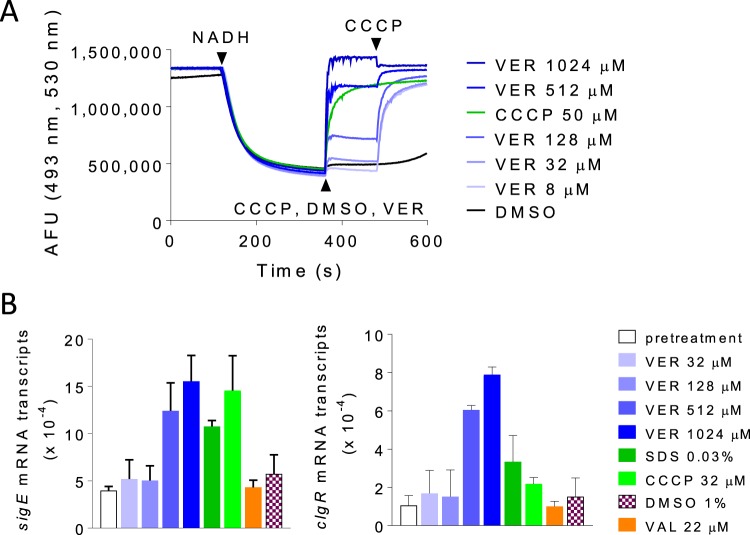
Effect of verapamil on membrane energetics and envelope stress. (A) Dose response of verapamil effect on proton translocation in inverted membrane vesicles of M. bovis BCG, followed by reversal performed with the protonophore CCCP. (B) Transcriptional induction of membrane stress reporters by verapamil in comparison to SDS and agents that perturb the proton motive force.

To examine the direct effect of verapamil on the mycobacterial membrane, we characterized the impact of the drug on purified inverted membrane vesicles (IMV) using acridine orange as a reporter of proton translocation. We measured a significant and concentration-dependent decrease in membrane ΔpH over the same range of verapamil concentrations in which drug potentiation could be observed (Fig. 6A). At concentrations that kill nonreplicating M. tuberculosis, verapamil exerted similar degrees of uncoupling to CCCP at 1× MIC (32 μM). At higher concentrations, the magnitude of this effect exceeded that achieved by CCCP and could be restored to the baseline precharged state upon subsequent addition of CCCP, suggesting that the effect of verapamil on membrane ΔpH may be mediated by a biological mechanism more complex than simple chemical uncoupling. In contrast, addition of valinomycin, which selectively dissipates the electrical component of the PMF, had no effect on verapamil-treated IMVs (Fig. S3), suggesting that verapamil had exerted a specific form of membrane stress that selectively impaired the ΔpH component of its PMF.

These results prompted us to investigate the effect of verapamil on genes corresponding to reporters of membrane stress, namely, *sigE* and *clgR*, the latter gene belonging to the cell envelope stress-sensing Psp system of M. tuberculosis (38). We found a dose-proportional increase in the levels of both *sigE* and *clgR* transcripts following exposure of growing cultures to verapamil for 1 h at concentrations similar to those required for M. tuberculosis growth inhibition and killing of nonreplicating M. tuberculosis bacteria. The level of the effect was comparable to or higher than that exerted by SDS and CCCP at 1× MIC (Fig. 6B). Similar results were obtained using a fluorescent transcriptional reporter corresponding to the *clgR* gene (Fig. S4). Thus, verapamil triggers induction of envelope-preserving functions in response to the membrane stress that it imposes on M. tuberculosis.

**FIG 7:**
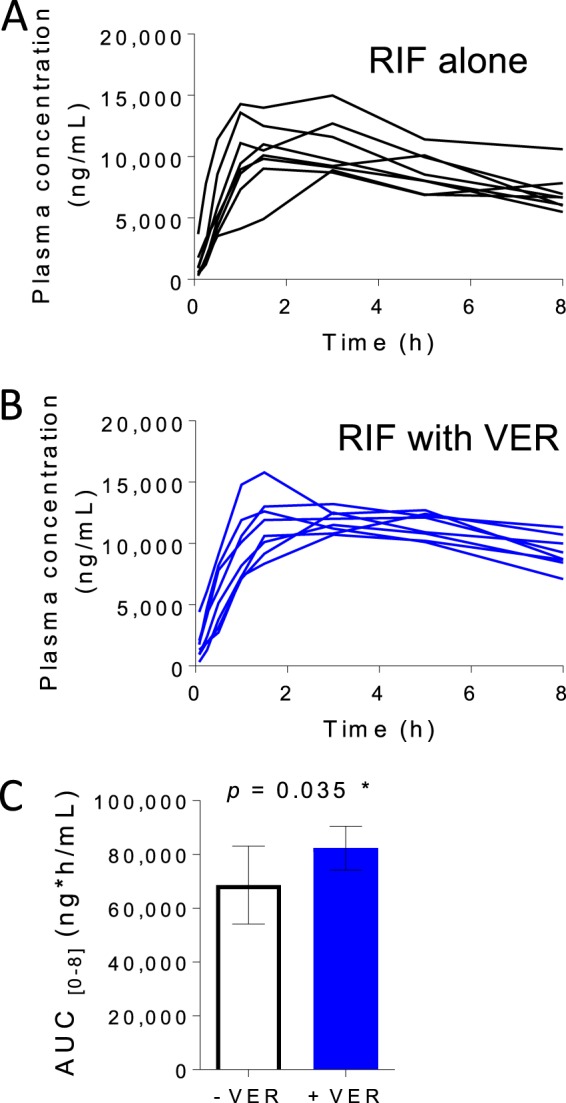
Effect of verapamil pretreatment on the pharmacokinetic parameters of rifampin (RIF) in CD-1 mice. Six-week-old female CD-1 mice were purchased from Charles River Laboratories (Wilmington, MA). Mice were dosed orally with 6.25 mg of verapamil/kg of body weight (area under the concentration-time curve from h 0 to h 8 [AUC_0–8_] = 255 ng · h/ml [human equivalent]) or vehicle (water) once daily for 8 days before a single oral dose of RIF was administered at 10 mg/kg on day 8. There were 8 mice in each treatment group. Blood was drawn by tail vein puncture at each time point, ranging from 0 to 8 h after administration of the RIF oral dose. Drug concentrations in mouse plasma were determined by liquid chromatography-mass spectrometry assays. (A) RIF plasma concentrations without verapamil pretreatment. Each line represents a single mouse. (B) RIF plasma concentrations with 7-day verapamil pretreatment. (C) Average AUC of RIF over 8 h with or without verapamil pretreatment (unpaired Student's *t* test).

In mice, verapamil administered at human-equivalent doses, which achieve concentrations significantly lower than its MIC, accelerated cure by the first-line drug regimen (19). Since verapamil inhibits P-glycoprotein, a eukaryotic drug efflux protein (4), and uptake transporters such as the OCT protein (5) that affect the absorption, distribution, metabolism, or elimination of drugs at various sites in the body (39), we hypothesized that the observed pharmacodynamic effect could have been due to pharmacokinetic drug-drug interactions. Indeed, transporter inhibition occurs at therapeutically achieved verapamil concentrations, and rifampin is a known substrate of these transporters. Accordingly, when we tested the effect of verapamil on rifampin's pharmacokinetic profile in the mouse, we observed a consistent increase in rifampin concentrations in blood under conditions in which verapamil had been predosed for 7 days (Fig. 7) (see Table S1 in the supplemental material). The results are consistent with modulation of the pharmacokinetic profile of rifampin through the inhibition of P-gp and OCT protein in liver cells, kidney cells, and other cells involved in drug disposition.

## DISCUSSION

We have shown that, in contrast to expectations, verapamil does not affect intracellular drug uptake and accumulation in M. tuberculosis through direct inhibition of efflux pumps. Similar results were obtained in two independent assays, thus excluding the possible presence of technical artifacts in the absence of a suitable positive control with demonstrated and direct efflux pump inhibitory activity in M. tuberculosis. Likewise, preincubation of human macrophage-like cell line THP-1 or activated murine bone marrow-derived macrophages with verapamil failed to increase the intramacrophage concentration of a range of anti-TB drugs, excluding the possibility that the potentiating effect of verapamil *in vivo* was due to accumulation of anti-TB drugs inside phagocytes. Instead, mechanistic investigations using physiologic and microbiologic readouts were consistent with a biologically specific impact of verapamil on M. tuberculosis membrane bioenergetics and with induction of a membrane stress response.

Verapamil has been shown to increase the intracellular accumulation of EtBr, a broad substrate of mammalian efflux pumps (33, 34) commonly used as a probe of efflux pump activity in bacteria (35), in M. tuberculosis (36). We have reproduced those results and found EtBr and verapamil to be synergistic. Rather than direct inhibition of efflux pumps by verapamil being responsible for the reported data, our results support an alternative explanation whereby verapamil-induced EtBr accumulation is an indirect consequence of altered efflux pump function arising from perturbed membrane energetics (34). In all drug uptake assays, the period of incubation with adjunctive verapamil had to be limited to 24 h in order to avoid artifacts associated with effects on the envelope integrity and culturability of M. tuberculosis on agar, which in turn affected the enumeration of CFU required to normalize measurements of intracellular drug concentrations. That was an intrinsic limitation of the present study.

With a pK_a_ of 8.92 and a cLogP (calculated partition coefficient between *n*-octanol and water) of 5.23, verapamil is a lipophilic weak base or a cationic amphiphile containing a tertiary amine which is protonated at physiological pH. These physicochemical properties suggest that verapamil may dissolve in lipid bilayers. Indeed, previous biophysical studies showed that verapamil inserts into artificial membranes and alters their electrical properties (40), consistent with our indirect observations that it associates with the mycobacterial membrane (see Fig. S5 in the supplemental material).

Interestingly, verapamil exerts its various effects within a wide range of concentrations that are largely consistent with the potentiation observed *in vivo* and *in vitro*. At the low micromolar concentrations achieved in patients and recapitulated in mice, we observed a pharmacokinetic interaction with rifampin leading to a boost in oral exposure over 8 h, likely due to inhibition of P-gp and other eukaryotic transporters known to be inhibited by verapamil. A similar effect was observed when rifampin was dosed to the steady state (41). Recently, Xu et al. showed that verapamil increases the efficacy of bedaquiline in mice to an extent that is the same as that to which it increases systemic bedaquiline exposure in plasma. They concluded that verapamil's adjunctive activity *in vivo* is likely due to enhanced systemic exposure to companion drugs via effects on mammalian transporters rather than to inhibition of bacterial pumps (42).

Dose-proportional effects on membrane energetics occur at a range of concentrations from 8 μM to 1 mM, consistent with the potentiation of bedaquiline and clofazimine *in vitro*. To exert growth inhibition or cidal activity on its own, verapamil must be used at a concentration of 512 μM or higher, which can be rationalized by the fact that the proton motive force is made up of the sum of two parameters, ΔΨ (electric potential) and ΔpH (transmembrane proton gradient), and that bacteria are thought to partially compensate for a decrease in the level of one by an increase in the level of the other (30, 31, 43). Thus, the PMF “buffering” capacity of bacteria may explain the difference between the concentrations required to affect membrane electric potential and those required to impair cell growth or viability. This compensation mechanism has been exploited in other bacterial species to discover combinations of perturbants that affect both components of the PMF (30).

Speculation notwithstanding, despite its canonical annotation as an inhibitor of mammalian efflux pumps, the foregoing data show that the potentiating activity of verapamil with antimycobacterial drugs *in vitro* need not be the consequence of increased intrabacterial drug accumulation but can instead be a consequence of the ability to directly impact membrane energetics. These findings thus shed fresh light on the therapeutic relevance of the membrane of M. tuberculosis as a pharmacologic target. Interestingly, several cationic amphiphilic anti-TB drugs, including SQ109, initially believed to exclusively target membrane proteins (the MmpL3 transporter in the case of SQ109), have since been found to interact with membrane lipids in a way that causes structural and electrical perturbations (26). In fact, due to its biophysical properties, the bacterial membrane may serve as an “enricher” for cationic amphiphiles (26). Recently, the use of membrane-inserting low-molecular-weight amphiphiles was specifically pursued as a novel antimycobacterial approach for therapy against both growing and nonreplicating bacteria notorious for their phenotypic resistance to conventional, growth-targeting antibiotics (27, 29). The ability of persistent organisms, including M. tuberculosis, to maintain an energized membrane in the face of prolonged quiescence is critical to their survival (23). Since the verapamil concentrations required to achieve bactericidal activity against M. tuberculosis are high and likely to be toxic to mammalian membranes, the design of more-potent and M. tuberculosis-specific verapamil analogs may be a valid approach to discover novel adjunct therapies that synergize with anti-TB drugs and that also directly target replicating and nonreplicating M. tuberculosis bacteria alike.

## MATERIALS AND METHODS

### Bacterial strains, culture conditions, and chemicals.

M. tuberculosis wild-type strain H37Rv (ATCC 27294), MDR strain R543 (7), and M. bovis BCG liquid cultures were grown in Middlebrook 7H9 broth (Sigma-Aldrich) supplemented with 0.5% albumin, 0.2% glucose, 0.085% sodium chloride, 0.2% glycerol, and 0.05% Tween 80. Solid cultures were grown on Middlebrook 7H11 agar (Sigma-Aldrich) supplemented with 0.5% albumin, 0.2% glucose, 0.085% sodium chloride, and 0.5% glycerol. M. tuberculosis strain Δ*panCD* Δ*leuD* liquid cultures were grown in Middlebrook 7H9 broth supplemented with 0.5% albumin, 0.2% glucose, 0.085% sodium chloride, 0.5% glycerol, 0.05% tyloxapol, 0.2% Casamino Acids, 24 μg/ml calcium pantothenate (Sigma-Aldrich), and 0.1 mg/ml l-leucine (Sigma-Aldrich). M. tuberculosis strain Δ*panCD* Δ*leuD* solid cultures were grown on Middlebrook 7H11 agar supplemented with 0.5% albumin, 0.2% glucose, 0.085% sodium chloride, 0.5% glycerol, 0.2% Casamino Acids, 24 μg/ml calcium pantothenate (Sigma-Aldrich), and 0.1 mg/ml l-leucine (Sigma-Aldrich).

Verapamil hydrochloride, pyrazinamide (PZA), HEPES, KCl, MgCl_2_, and succinic acid were purchased from Fisher Scientific. Tyloxapol, l-leucine, calcium pantothenate, bedaquiline, acetyl isoniazid, linezolid (LZD), clofazimine, hygromycin B, acridine orange, and oxonol VI were purchased from Sigma-Aldrich. Rifampin was purchased from Gold Biotechnology, USA. Isoniazid was purchased from Fagron. Moxifloxacin (MXF) hydrochloride was purchased from Chemieliva Pharmaceutical, China. DiSC_3_(5) (3,3′-dipropylthiadicarbocyanine iodide) was purchased from Thermo Fisher Scientific. Valinomycin and CCCP were purchased from Tocris. Sytox green nucleic acid stain and ethidium bromide were purchased from Invitrogen.

### MIC measurements and determination of synergy.

Initial stock solutions of compounds were made in dimethyl sulfoxide (DMSO), and further dilutions were prepared from those solutions. All strains were grown to the logarithmic phase and diluted in broth to an optical density (OD) of 0.02. Two-fold serial dilutions of compounds were prepared in DMSO and dispensed into 96-well microtiter plates. A 198-μl volume of diluted cultures was added to 96-well microtiter plates. The microplates were sealed and incubated at 37°C for 5 days. To avoid interference with the solvent, the concentration of DMSO was kept constant at 1%, a level which does not affect M. tuberculosis growth. The OD at 600 nm (OD_600_) was measured in a microplate reader (BioTek), and growth inhibition data were calculated. For MIC measurements at different pH values, 7H9 complete medium was adjusted with HCl or NaOH to pH 6.0 or pH 7.5. Logarithmic-phase cultures were resuspended in media with different pH values and seeded into microtiter plates.

The levels of synergy between verapamil and rifampin, bedaquiline, or clofazimine against H37Rv and R543 were determined using the checkerboard method. To avoid interference with the solvent, the concentration of DMSO was kept constant at 2%, which is a level that does not affect M. tuberculosis growth. Two-fold serial dilutions of compounds were prepared in DMSO and dispensed into 96-well microtiter plates in an 8-by-8 square format. H37Rv and R543 were grown to the logarithmic phase and diluted in broth to an optical density of 0.02. A 196-μl volume of diluted cultures was added to 96-well microtiter plates. The microplates were sealed and incubated at 37°C for 5 days. The optical density (OD_600_) was measured in a microplate reader (BioTek), and levels of growth inhibition were calculated. Levels of growth inhibition of more than 90% were recorded as representing no bacterial growth. The FIC (fractional inhibitory concentration) of each drug was calculated as the MIC of the drug used in a combination divided by the MIC of the drug used alone (MIC_[drug A+B]_/MIC_[drug A]_). The FIC index (FICI) value represents the sum of FIC_[drug A]_ + FIC_[drug B]_. FICI values of ≤0.5 indicate synergy, FICI values of ≥4 indicate antagonism, and 0.5<FICI<4 values indicate additivity or indifference (44, 45).

### Susceptibility testing on solid medium.

Experimental MICs were determined in 24-well plates by using the proportion method as described by Wallace et al. (46) with some modifications. Rifampin (Sigma) was adjusted in 7H10 medium (Difco) supplemented with 0.2% glycerol, 0.04% (0.5 g/liter) bovine serum albumin (BSA), and 0.085% NaCl to final concentrations of 0.006 to 7.2 μg/ml. Ten microliters of a M. tuberculosis H37Rv bacterial inoculum at an OD_600_ of 1 (5 × 10^8^ cells per ml) was inoculated on 7H10 medium containing increasing concentrations of rifampin and/or verapamil (60 μg/ml [132 μM]), followed by incubation at 37°C for 3 weeks. The minimal antibiotic concentration that completely suppressed bacterial growth was designated the experimental MIC.

### Drug penetration assay in Mycobacterium tuberculosis.

Drug penetration assays were performed in M. tuberculosis Δ*panCD* Δ*leuD* essentially as reported before (47). Briefly, M. tuberculosis Δ*panCD* Δ*leuD* cells were grown to mid-logarithmic phase and harvested by centrifugation (5,200 rpm; 10 min; 4°C). The pellets were then resuspended in appropriate volumes of fresh growth medium and adjusted to an OD_600_ of 4.0. CFU were enumerated on agar plates. Cultures were preincubated for 3 min with VER at 128 μM (0.25 MIC) prior to addition of drugs. After 30 min of incubation with drugs with gentle agitation (500 rpm), triplicate 300-μl samples were removed and pelleted by centrifugation (15,000 rpm; 5 min; 4°C) and washed twice with equal volumes of ice-cold phosphate-buffered saline (PBS) with Tween 80. Bacilli were then resuspended in an equal volume of 0.1 M glycine–HCl (pH 3.0), incubated overnight at 37°C under conditions of gentle agitation (500 rpm), and finally disrupted by sonication for 1 min. Lysates were cleared by centrifugation (15,000 rpm; 5 min; room temperature) and sterilely filtered twice (Millipore Millex-GV polyvinylidene difluoride [PVDF] membrane; 0.22-μm pore size; 13-mm diameter). Compound extraction was achieved by adding 80 μl of methanol and 40 μl of acetonitrile. Lysate samples were subsequently stored at −20°C or analyzed immediately. The drugs were present as follows: rifampin (RIF) at 0.9 μM (0.74 μg/ml), isoniazid (INH) at 53 μM (7.27 μg/ml), pyrazinamide (PZA) at 100 μM (12.31 μg/ml), moxifloxacin (MXF) at 1 μM (0.4 μg/ml), linezolid (LZD) at 10 μM (3.37 μg/ml), bedaquiline (BDQ) at 0.03 μM (0.017 μg/ml), and clofazimine (CFZ) at 0.5 μM (0.24 μg/ml). These concentrations correspond to either 0.25× MIC of each drug or the lowest concentration required to reach the limit of quantitation by HPLC coupled to mass spectrometry, whichever was lower.

For the drug penetration assays performed with M. tuberculosis H37Rv and R543, cells were grown to mid-logarithmic phase and plated on agar plates to enumerate CFU levels. Cultures were then divided into aliquots in polystyrene tubes and preincubated for 3 min with VER at 128 μM (1/4 MIC) prior to addition of drugs. After 30 min of incubation with CFZ (4 μM) and RIF (0.015625 μM) with gentle agitation, samples were pelleted twice by centrifugation (6,000 × *g*; 5 min; 4°C). Supernatants were then carefully collected and stored in polystyrene tubes at −20°C or analyzed immediately.

For the VER penetration assay in M. tuberculosis H37Rv, cells were grown to mid-logarithmic phase and harvested by centrifugation (5,200 rpm; 10 min; 4°C). The pellets were then resuspended in appropriate volumes of fresh growth medium and adjusted to an OD_600_ of 4.0. CFU levels were enumerated on agar plates. Cultures were incubated for 30 min with VER at 128 μM (0.25 MIC). After 30 min of incubation with gentle agitation, triplicate 300-μl samples were removed and pelleted by centrifugation (15,000 rpm; 5 min; 4°C) and washed twice with equal volumes of ice-cold PBS with Tween 80. Bacilli were then resuspended in an equal volume of 0.1 M glycine–HCl (pH 3.0), incubated overnight at 37°C under conditions of gentle agitation, and, finally, disrupted by sonication for 1 min. Lysates were cleared by centrifugation (15,000 rpm; 5 min; room temperature), and the supernatant was collected and split into two parts. One part of the supernatant was sterilely filtered twice, and the other part of the supernatant was left unfiltered. Compound extraction was done as described above. Lysate samples were subsequently stored at −20°C or analyzed immediately.

The measurement on a real-time basis of ethidium bromide (EtBr) uptake by the M. tuberculosis H37Rv strain was performed using a fluorometric method described previously (37) with some minor modifications. Briefly, H37Rv was grown to mid-logarithmic phase and diluted in growth medium to a final OD_600_ of 0.05. A 196-μl volume of this diluted culture was divided into aliquots that were placed into black-sided clear-bottom 96-well plates. A 2-μl volume of DMSO or VER was added to wells, and fluorescence was read at 590 nm (excitation wavelength [Ex], 530 nm) for 3 min at 37°C under shaking conditions. Then, 2 μl EtBr was added and fluorescence was read for 24 h at 37°C under shaking conditions.

### M. tuberculosis filter culture and extraction of metabolites.

M. tuberculosis H37Rv was cultured in a biosafety level 3 facility at 37°C in 7H9 broth (Difco) or 7H10 broth (Difco) supplemented with 0.2% glycerol, 0.04% tyloxapol, 0.5 g/liter BSA, and 0.085% NaCl. M. tuberculosis-laden filters were generated for use in metabolomic profiling as previously described and were incubated at 37°C for 5 days to reach the mid-logarithmic phase of growth (48). The M. tuberculosis-laden filters were transferred on day 6 to plastic insertions containing rifampin (10 μM), bedaquiline (5 μM), isoniazid (5 μM), ethambutol (5 μM), or clofazimine (1 μM) in combination with verapamil (60 μg/ml [132 μM]) in liquid medium in direct contact with the underside of the bacteria-laden filter for 24 h. Next, the M. tuberculosis-laden filters were extracted and subjected to liquid chromatography-mass spectrometry as previously described (49).

### Drug penetration assays in human THP-1 cells.

Drug penetration assays in human THP-1 cells were performed as previously reported (50). THP-1 cells (ATCC TIB-202) were initiated at a density of 2 × 10^5^ to 4 × 10^5^ cells/ml in flasks in RPMI 1640 medium (Corning) supplemented with 10% fetal bovine serum and 2 mM l-glutamine (Sigma, St. Louis, MO). After 3 days of incubation, viable cells were counted using the trypan blue exclusion method and diluted to 6.67 × 10^5^ cells/ml. Phorbol 12-myristate 13-acetate (PMA) was added to reach a final concentration of 100 nM, and 1 × 10^5^ cells were seeded into each well of 96-well tissue culture-treated plates (Greiner Bio One, Monroe, NC). After overnight incubation, culture medium was carefully removed and media containing drugs (RIF at 4 μM [3.29 μg/ml]; INH at 30 μM [4.11 μg/ml]; PZA at 4 mM [492 μg/ml]; MXF at 5 μM [2 μg/ml]; LZD at 30 μM [10.12 μg/ml]; BDQ at 0.5 μM [0.28 μg/ml]; CFZ at 1 μM [0.47 μg/ml]) were added with or without VER at 128 μM. After 30 min of incubation in an ambient environment, media were removed and cells were gently washed twice with an equal volume of ice-cold PBS to remove any extracellular drug residuals. Cells were lysed with an equal volume of deionized water for 1 h at 37°C in an ambient environment. Lysates were transferred to 1.5-ml centrifuge tubes and stored at −20°C or analyzed immediately. To quantify the total number of cells/well, 50 μl of each cell lysate was added to a clear-bottom black-sided 96-well plate. A 50-μl volume of deionized water and 100 μl of PicoGreen (Life Technologies) were added, and the plates were incubated for 2 to 5 min under conditions of protection from light. Fluorescence was read at 520 nm (excitation wavelength, 480 nM). Readings were normalized, and cell number interpolations were made from a standard curve.

### Calculation of intracellular concentrations in drug uptake assays.

The method of calculation of cellular concentrations was adapted from a previous study (47). In instances in which levels of drug accumulation in various penetration assays were compared, values corresponding to the drug concentrations in cell lysates were multiplied by the value corresponding to the final volume (lysate plus organic solvents). The result represented an expression of intracellular uptake relative to a no-VER control. Given the average bacillus width of 0.25 μm and bacillus lengths ranging from 1.5 to 4 μm for mycobacteria (51, 52), the estimated average cell volume of 0.5 μm^3^ was used for the calculation of intracellular drug concentrations (expressed in micromoles) for drug accumulation in M. tuberculosis. The average THP-1 cell diameter of 11.3 μm was measured by confocal microscopy and the calculated average cell volume of 755.5 μm^3^ was used for the calculation of intracellular drug concentrations (expressed in micromoles) for drug accumulation in THP-1 cells. For all uptake assays, the illustrated means represent the averages of results from three technical replicates. Standard deviations are shown as error bars. Intracellular concentration/extracellular concentration (IC/EC) ratios were also calculated by normalizing for the drug incubation concentration.

### Quantification of drugs in cell lysates.

All bacterial and macrophage lysate samples were extracted with organic solvent as mentioned above. Five microliters of 75 mg/ml ascorbic acid was added to the RIF standards and the study samples before extraction to prevent auto-oxidation and conversion to RIF-quinone at physiological pH.

Quantification of drug concentrations was achieved by liquid chromatography-tandem mass spectrometry (53). LC/MS-MS analysis was performed on a Sciex Applied Biosystems Qtrap 4000 triple-quadrupole mass spectrometer coupled to an Agilent 1260 HPLC system to quantify each drug in the samples. PZA chromatography was performed with an Agilent Zorbax SB-C8 column (4.6 by 75 mm; particle size, 3.5 μm) using a reverse-phase gradient elution. INH, MXF, RIF, RIF, BDQ, LZD, VER, and CFZ chromatography was performed on an Agilent Zorbax SB-C8 column (2.1 by 30 mm; particle size, 3.5 μm) using a reverse-phase gradient elution. Acetyl-INH chromatography was performed on a Cogent Diamond Hydride column (2.1 by 50 mm; particle size, 4 μm) using a normal phase gradient. All gradients used 0.1% formic acid–Milli-Q deionized water for the aqueous mobile phase and 0.1% formic acid–acetonitrile for the organic mobile phase. Multiple-reaction monitoring of parent-daughter transitions in electrospray positive-ionization mode was used to quantify the analysts. Data processing was performed using Analyst software (version 1.6.2; Applied Biosystems Sciex).

### Time-kill assays.

M. tuberculosis H37Rv was grown to the logarithmic or stationary phase and treated with VER at increasing concentrations (64 to 512 μM). Samples were removed at various time points for CFU determinations on agar plates. The DMSO concentration was kept constant (1%) in all samples.

Time-kill assays against nutrient-starved nonreplicating M. tuberculosis were performed as previously reported (23, 54). Exponentially growing M. tuberculosis H37Rv bacilli were harvested by centrifugation (3,200 rpm; 4°C; 5 min), washed twice with PBS supplemented with 0.025% Tween 80, and diluted to a final OD_600_ of 0.2. The resultant culture diluted to an OD_600_ of 0.2 was starved for 14 days (37°C; 150 rpm). Clumps were removed by centrifugation (200 × *g*; 3 min; room temperature) before addition of drugs (VER at 1,024 and 512 μM; INH at 32 μM [16× MIC]; RIF at 8 μM [1,024× MIC]). Samples were taken at various time points for CFU determinations on 7H11 solid medium. The DMSO concentration was kept constant (1%) under all conditions.

### Membrane potential assay.

Membrane depolarization was monitored using fluorescent probe 3,3′-dipropylthiadicarbocyanine iodide [DiSC_3_(5)], which partitions into the plasma membrane in proportion to the membrane potential (55). Dissipation of the membrane potential releases the probe, leading to an increase in fluorescence. Exponentially growing M. tuberculosis H37Rv bacilli were harvested by centrifugation (10,000 relative centrifugal force [rcf]; 25°C; 5 min), washed once with buffer containing 5 mM HEPES and 5 mM dextrose (pH 7.2) (buffer A), and resuspended in the same buffer to an OD_600_ of 0.05. Fluorescence was monitored (excitation wavelength [Ex], 622 nm; emission wavelength [Em], 670 nm) before addition of DiSC_3_(5) (and KCl for the valinomycin control) to subtract autofluorescence background. Then, 2 μM DiSC_3_(5) was added, and cultures were dispensed into black-sided clear-bottom plates (200 μl/well). Cells were equilibrated in the presence of the dye for 2 h to allow dye uptake into the lipid bilayer and fluorescence self-quenching, resulting from aggregation of the dye within the lipid bilayer, prior to addition of drug and fluorescence measurements. Fluorescence was monitored (excitation wavelength, 622 nm; emission wavelength, 670 nm) on a BioTek fluorescence microplate reader at 37°C. Then, drugs were added and fluorescence was read (excitation wavelength, 622 nm; emission wavelength, 670 nm) immediately for 1 h at 37°C. Wells with cells, DiSC_3_(5), and KCl served as controls.

### Cell content release assay.

To assess the effect of VER on the release of cell content, a M. tuberculosis Δ*panCD* Δ*RD1* (Hyg^r^) strain with plasmid pFPV27 under the control of a Mycobacterium strong promoter (MSP) expressing a red fluorescent protein (tdTomato) (kindly provided by Lalita Ramakrishnan) was used. The plasmid was derived from pMSP12::GFP (green fluorescent protein) by interrupting the *aph* gene (removing a small [∼300-bp] Nsil fragment) and inserting the gene for hygromycin resistance. The GFP was then replaced with tdTomato. The tdTomato open reading frame (ORF) is present at bp 102 to bp 1532. The strain was grown to the logarithmic phase, and drugs were added (VER at 2,048 μM, 512 μM, and 128 μM; VAL at 22 μM; INH at 2 μM). Cultures were incubated at 37°C with gentle shaking. Samples were taken at various time points (4 h, 24 h, and 48 h), and the supernatant was collected by two serial centrifugations (6,000 × *g*; 5 min; 4°C) (56). Mechanical breakage by bead beating was used for disrupting M. tuberculosis cells. Zirconia/silica beads (0.1-mm diameter) were added to microcentrifuge tubes to reach approximately 1/6 full. Bacterial samples were then added to tubes such that the tubes were no more than half full. The cells were then homogenized (6.5 m/s) for 3 min. Fluorescence was measured at 554 nm (excitation) and 581 nm (emission).

### Sytox green uptake assay.

Bacterial cytoplasmic membrane permeation was monitored using a Sytox green uptake assay (57). Sytox green is a cationic cyanine dye (∼900 Da) that is not membrane permeative. When a cell's plasma membrane integrity is compromised, influx of the dye and subsequent binding to DNA cause a large increase in fluorescence. For the Sytox green assays, M. tuberculosis H37Rv bacilli were grown to the mid-exponential-growth phase (OD_600_ of 0.6) and then centrifuged (3,200 rpm; 5 min; room temperature), washed twice with 20 mM PBS, and resuspended in 20 mM PBS at an OD_600_ of 0.1. Cells were incubated with 3 μM Sytox green for 15 min in the dark at 37°C prior to the influx assay. Background fluorescence (Ex, 497 nm; Em, 523 nm) was measured for 3 min before addition of drugs (VER at 2,048 μM, 512 μM, and 128 μM; VAL at 22 μM; INH at 2 μM), and the increase in Sytox green fluorescence was measured for 1 h at 37°C. Bacilli lysed with bead beating were used as a positive control (using the procedure described above).

### Effect of verapamil on electric potential and ΔpH in M. bovis BCG inverted membrane vesicles.

M. bovis BCG inverted membrane vesicles (IMVs) were prepared using a previously described cell fractionation protocol (56). Proton translocation into IMVs was measured by monitoring the fluorescence of an acridine orange (AO) probe (57) using a PTI (Photon Technology International) fluorescence spectrophotometer. The assay buffer contained 10 mM HEPES (pH 7.5), 100 mM KCl, 5 mM MgCl_2_, 0.05 mg/ml of IMVs, and 5 μM AO. The membrane vesicles were energized with 200 μM NADH. Once equilibrium was reached, verapamil or a control solution (50 μM CCCP or DMSO only) was added as indicated. Once a plateau was achieved, 50 μM CCCP or 1 μM valinomycin was added. The excitation and emission wavelengths were 493 and 530 nm, respectively.

### Real-time quantitative reverse transcription-PCR assay.

For gene expression analyses, mid-log cultures of M. tuberculosis H37Rv were treated with VER at 32, 128, 512, and 1,024 μM, SDS at 0.03%, valinomycin at 22 μM, CCCP at 32 μM, and DMSO at 1% for 1 h. Aliquots of 1.8 ml of culture were collected and harvested by centrifugation (13,400 rpm; 45 s; room temperature). Bacterial cell pellets were resuspended in 1 ml TRIzol reagent (Invitrogen, Carlsbad, CA, USA), and 0.8 ml zirconia/silica beads (0.1-mm diameter) was added. Cells were disrupted in a bead beater by the use of three 45-s pulses with 10 min of incubation on ice between pulses. Cells were lysed by addition of 100 μl BCP reagent (Molecular Research Center) and vigorous mixing for 2 to 3 min. After 10 min of incubation at room temperature, the tubes were centrifuged (14,000 rpm; 30 min; 4°C). A 360-μl volume of the aqueous phase was transferred to fresh tubes containing 360 μl isopropanol for overnight precipitation at −20°C. After four cycles of overnight precipitation, samples were washed with 75% ethanol, air dried, and resuspended in nuclease-free water for storage at −80°C. Reverse transcription was performed with random hexameric primers and ThermoScript reverse transcriptase. Enumeration of mRNA transcripts was carried out by quantitative PCR (qPCR) using gene-specific primers, molecular beacons, and AmpliTaq Gold polymerase in a Stratagene Mx4000 thermal cycler.

### P*clgR*-mCherry induction assay.

The M. bovis BCG P*clgR*-mCherry reporter strain and the promoter induction assay have been described previously (29). Seed stocks of the reporter strain were grown to mid-log phase (OD_600_ = 0.4 to 0.6) and diluted to a starting OD_600_ of 0.1. A 100-μl volume of culture was added to 96-well microtiter plates containing 100 μl Middlebrook 7H9 medium with drugs at 0.5×, 1×, and 2× MIC_90_ to give a final volume of 200 μl per well. After measurement of fluorescence at time point 0, the plates were incubated at 37°C with shaking at 80 rpm for 24 h. Fluorescence was measured using an Infinite M200Pro plate reader (Tecan), and data were recorded as relative fluorescence units (RFU) (λ_ex_ = 587 nm/λ_em_ = 630 nm).

### Statistical analysis.

All statistical comparisons were performed with unpaired two-sample *t* tests. Statistical significance was set at a *P* value of <0.05.

## Supplementary Material

Supplemental material
