# Supplementary material for: Verapamil Targets Membrane Energetics in Mycobacterium tuberculosis
Source: Antimicrob Agents Chemother. 2018 Apr 26;62(5):e02107-17. doi: 10.1128/AAC.02107-17 (PMC5923092; doi:10.1128/AAC.02107-17)
Supplement: Supplemental material [file supp_62_5_e02107-17__index.html]

Supplemental material 

# Verapamil Targets Membrane Energetics in Mycobacterium tuberculosis

## Supplemental material

- Supplemental file 1 -

  Fig. S1 to S5 and Tables S1 and S2

  PDF, 742K
