# Supplementary material for: Verapamil Targets Membrane Energetics in Mycobacterium tuberculosis
Source: Antimicrob Agents Chemother. 2018 Apr 26;62(5):e02107-17. doi: 10.1128/AAC.02107-17 (PMC5923092; doi:10.1128/AAC.02107-17)
Supplement: Supplemental material [file AAC.02107-17_zac005187106s1.pdf]

Table S1. Effect of pre-dosing verapamil at 6.25 mg/kg (the human equivalent dose) once daily for 8 days on the pharmacokinetic parameters of rifampicin (RIF) dosed orally (p.o.) once on day 8 at 10 mg/kg. Values are means ± SD (*n*=8).

Pharmacokinetic parameters	RIF 10 mg/kg p.o.	
	control	Verapamil 6.25 mg/kg
C_{max} (ng/mL)	11,268 ± 2,256	12,600 ± 1,499
AUC_{0-8} (ng.h/mL) of verapamil	NA	255
elim $T_{1/2}$ (h)	8.4	13.6
AUC_{0-8} or rifampicin (ng.h/mL)	68,630 ± 14,533	82,359 ± 8,128* (*p*=0.035)

*Significant difference ($p < 0.05$) in comparison with the control (unpaired Student's *t*-test).

Table S2. PCR primers and molecular beacons used in this study

Genes	Primer pairs (5'-3') and Molecular beacon (MB)
clgR	Forward: TGAGCCTCGGGTATCTGTCGG<br>Reverse: AAGGCGCTCTTGACGCGCCAT<br>MB: ACGGGGCGAGCTGCTCAGTGCGATTTGTACCCCGT
sigE	Forward: ACGACTTGCCAACTTATTGCAG<br>Reverse: GGATGAGACATGCTGGTCGGA<br>MB: CGCACGATATCACGACCATCACGACCTTGCGTGCG
16s *rRNA*	Forward: ATGACGGCCTTCGGGTTGTAA<br>Reverse: CGGCTGCTGGCACGAGTTT<br>MB: CCCCGCCGACGAAGGTCCGGGTTCTCGCGGGG

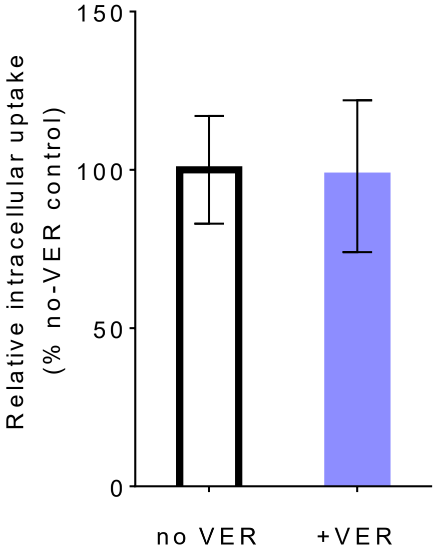

Figure S1. Effect of verapamil (VER) on the intracellular concentration of CFZ in *M. tuberculosis* H37Rv over 24 h. Cells were grown to mid-logarithmic phase and plated on Middlebrook 7H11 to enumerate colony forming units (CFU). Cultures were then aliquoted into polystyrene tubes and pre-incubated for 3 min with DMSO or verapamil at 128 µM (1/4 MIC) prior to addition of clofazimine. After 24 h incubation with clofazimine 4 µM with gentle agitation, a fraction of the samples was plated on 7H11 to enumerate CFU and the remainder was pelleted twice by centrifugation (6,000 g; 5 min; 4 °C). Supernatants were then carefully collected and stored in polystyrene tubes at -20 °C or analyzed immediately. Each experiment includes technical duplicates and was performed three times independently. Data from one representative experiment are shown. $p > 0.05$ (0.9125)

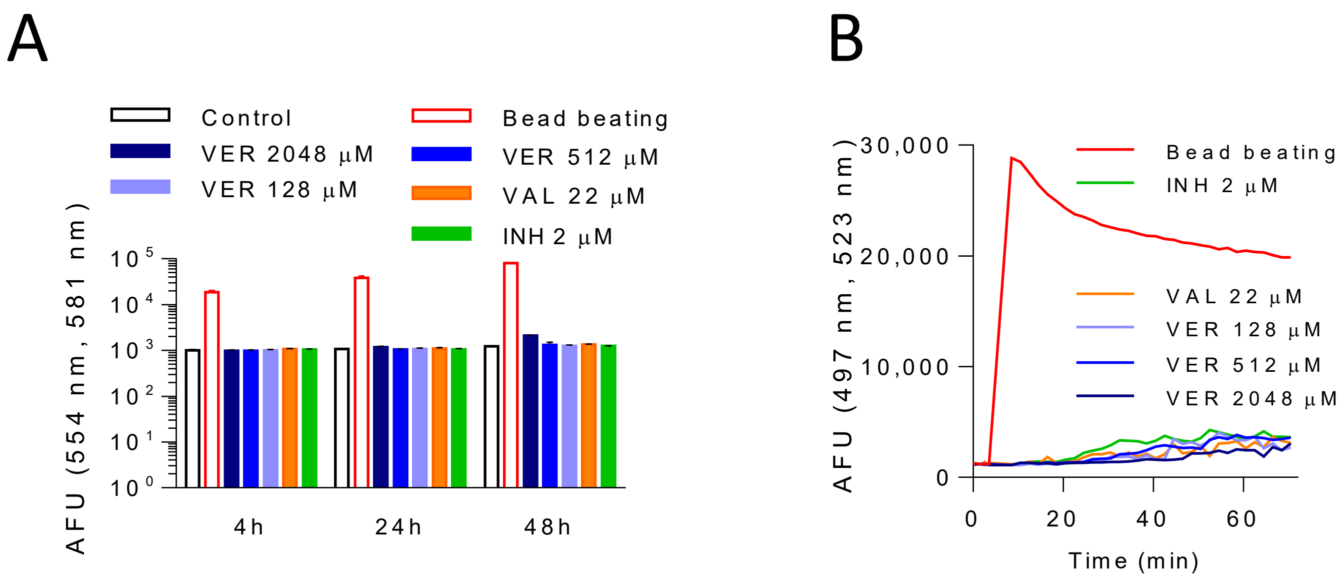

Figure S2: Effect of verapamil on membrane permeability to small and large molecules **(A)** Effect of verapamil on cytoplasmic protein leakage. Exponentially growing *M. tuberculosis ΔpanCD ΔRD1* (Hyg^R) (harboring plasmid pFPV27 expressing a red fluorescent protein (tdTomato, RFP, λ 581 nm emission / 554 nm excitation) under the mycobacterium strong promoter [MSP]) was exposed to increasing verapamil concentrations for 48 h, after which release of the red fluorescent protein was measured in the supernatant. Beat beating (BB) and the valinomycin (VAL) ionophore in the presence of exogenous K^+ were used as positive and negative controls, respectively. **(B)** Membrane permeability to small molecules was determined by monitoring intracellular accumulation of the non-membrane permeable cationic cyanine dye Sytox Green after incubation of *M. tuberculosis* culture with verapamil. Each experiment includes technical triplicates and was performed twice independently. Data from one representative experiment are shown in each panel. Verapamil did not result in increased release of a red fluorescent protein or increased permeability to Sytox Green, compared to drug-free control.

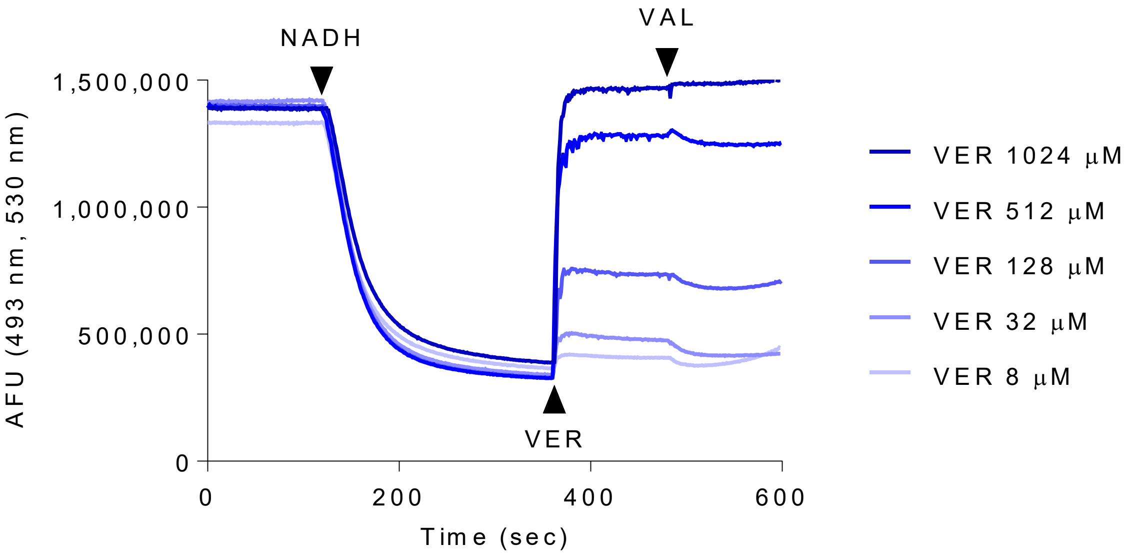

Figure S3: Effect of verapamil on proton translocation was measured in *M. bovis* BCG inverted membrane vesicles as monitored by measuring the fluorescence of acridine orange using a PTI (Photon Technology International) fluorescence spectrophotometer. The assay buffer contained 10 mM HEPES (pH 7.5), 100 mM KCl, 5 mM $MgCl_2$, 0.05 mg/mL of IMVs and 5 µM AO. The membrane vesicles were energized with 200 µM NADH. Once equilibrium was reached, verapamil or control solutions (50 µM CCCP or DMSO only) were added as indicated. Once a plateau was achieved, 50 µM CCCP or 1 µM valinomycin (VAL) were added. The excitation and emission wavelengths were 493 and 530 nm, respectively. As expected, VAL did not revert the effect of verapamil, since it affects membrane potential but not proton translocation.

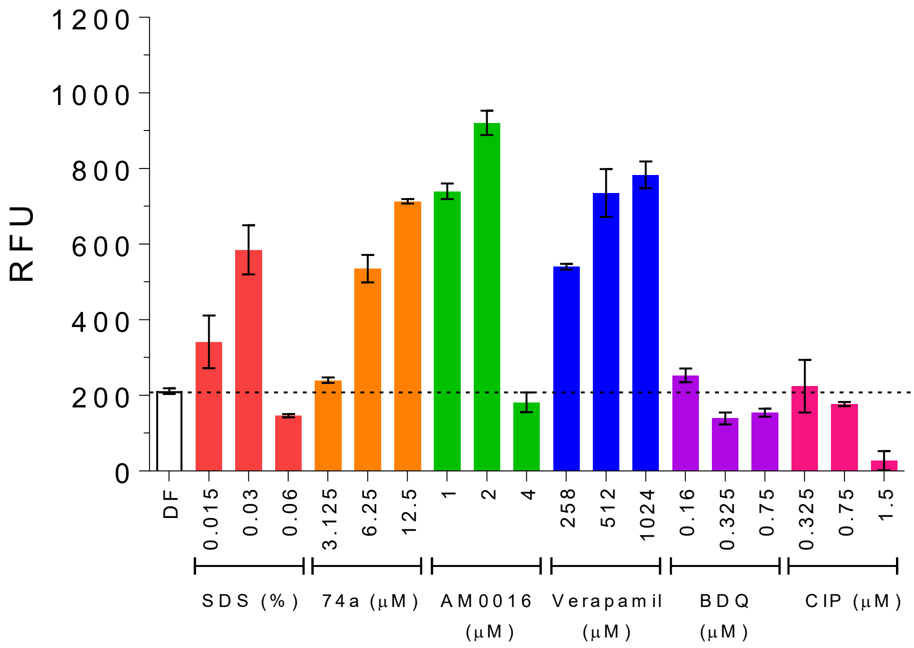

Figure S4: Induction of the *clgR* promoter by various membrane-active compounds. Cultures of *Mycobacterium bovis* BCG carrying a p*clgR*-mCherry transcriptional fusion were treated with various agents at 1/2x, 1x and 2x MIC_{90} for 24 h and Relative Fluorescence Units (RFU) were measured. DF, drug free control. Positive controls: SDS, sodium dodecyl sulfate (1); 74a, a membrane inserting amphiphilic indole based lead compound (2), AM0016, a membrane inserting xanthone (3). Negative controls: BDQ, ATP synthase inhibitor bedaquiline; CIP, gyrase inhibitor ciprofloxacin. The experiments were carried out three times independently and mean values and standard deviations are shown. Note: SDS, AM0016 and CIP at the highest concentration resulted in a drop of RFU, likely due to the cidal activity of these agents observed at 2x MIC (1, 3).

Description of the results: Recently it was shown that the promoter of the transcription factor gene *clgR* (part of the cell envelope stress-sensing Psp system of *Mycobacterium tuberculosis* (2)), is induced by various membrane inserting agents, including SDS (2) and a novel amphiphilic indole based lead compound 74a (1). To provide further evidence for membrane stress sensitivity of p*clgR* activity we tested another recently identified membrane inserting lead compound, the xanthone derivative AM0016 (3) and again observed dose dependent induction of p*clgR* promoter activity. In contrast, drugs not interfering with membrane integrity such as the ATP synthase inhibitor bedaquiline or the gyrase inhibitor ciprofloxacin did not cause increased activity of the promoter. **Fig. S4** shows that verapamil treatment also induced p*clgR* activity in a dose dependant manner, similar to the membrane inserting lead compounds 74a and AM0016, consistent with a membrane integrity disrupting function of verapamil.

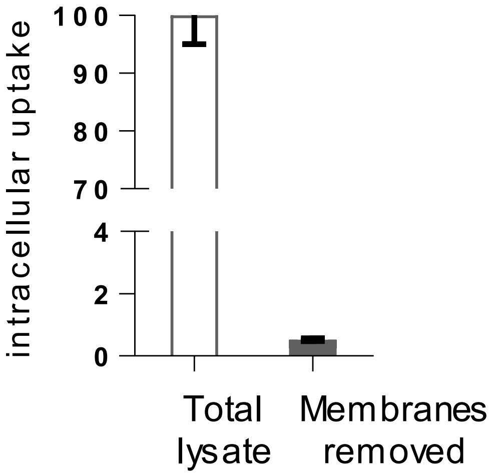

Figure S5. Partitioning of verapamil in the membrane fraction of Mtb. Total concentrations of bacteria-associated verapamil were measured prior to and following removal of the membrane fraction. The intrabacterial uptake of verapamil into *M. tuberculosis* yields a cell-associated/extracellular ratio of 22.4 (± 2.0) when verapamil concentration is measured in whole cell lysates. Following removal of cell wall and membrane debris by filtration, the lysate concentration drops to 0.52% (± 0.044%) of the total cell-associated concentration (two tailed unpaired *t*-test was used with $p = 0.0008$).

REFERENCES

1. Datta P, Ravi J, Guerrini V, Chauhan R, Neiditch MB, Shell SS, Fortune SM, Hancioglu B, Igoshin OA, Gennaro ML. 2015. The Psp system of Mycobacterium tuberculosis integrates envelope stress-sensing and envelope-preserving functions. Mol Microbiol 97:408-22.
2. Yang T, Moreira W, Nyantakyi SA, Chen H, Aziz DB, Go ML, Dick T. 2017. Amphiphilic Indole Derivatives as Antimycobacterial Agents: Structure-Activity Relationships and Membrane Targeting Properties. J Med Chem 60:2745-2763.
3. Mukherjee D, Zou H, Liu S, Beuerman R, Dick T. 2016. Membrane-targeting AM-0016 kills mycobacterial persisters and shows low propensity for resistance development. Future Microbiol 11:643-50.
